# Hybrid Telehealth Adaptation of COMPASS for Hope: Parent-Mediated Outcomes in Autism

**DOI:** 10.3390/bs15111561

**Published:** 2025-11-15

**Authors:** Alexis D. Rodgers, Brittany A. Dale, Lisa A. Ruble

**Affiliations:** 1 Behavioral Science Division, UPMC Children’s Hospital of Pittsburgh, Pittsburgh, PA 15224, USA; rodgersad2@upmc.edu; 2 Department of Special Education, Ball State University, Muncie, IN 47306, USA; laruble@bsu.edu

**Keywords:** autism, behavior, telehealth, parent-mediated intervention, asynchronous intervention, parent stress, parenting sense of competency

## Abstract

There are limited empirically supported interventions that target three outcomes—behavior of children with ASD (instead of using adjectives such as “disruptive,” “interfering,” “problem,” or “challenging” behavior, we use “behavior” to avoid ableist language), parent stress, and parenting sense of competence. To help address this need, we tested a hybrid telehealth adaptation of COMPASS for Hope (C-HOPE), an 8-week parent-mediated program originally offered via face-to-face or synchronous telehealth delivery. The present study incorporated asynchronous group discussion board sessions hosted on a learning-management platform together with synchronous individual coaching sessions by telephone. Using a pre-post design, 10 caregivers completed the intervention. Effect sizes were calculated for three treatment outcomes of child behavior, parent stress, and parenting sense of competence. There was a statistically significant difference in the scores for child behavior, with a large effect size (*d* = 0.73) and a statistically significant difference in parent stress, with a medium effect size (*d* = 0.50). No difference was observed for parenting sense of competence. Treatment adherence and caregiver satisfaction for the intervention were acceptable. Findings support the feasibility and promise of combining asynchronous and synchronous telehealth elements to increase access to evidence-based parent-mediated interventions for ASD.

## 1. Introduction

Parenting competency and stress and child behavior can be salient issues for many families but especially notable for families of children with autism spectrum disorder (ASD). Although behaviors such as aggression and self-injury are not included in the diagnostic criteria outlined in the *Diagnostic and Statistical Manual of Mental Disorders, Fifth Edition, Text Revision* (DSM-5-TR; American Psychiatric Association, 2022), researchers have consistently reported higher levels of these behaviors in individuals with ASD even compared to other children with developmental disorders (e.g., Adams et al., 2019; Chandler et al., 2016). Furthermore, these behaviors are not isolated to the child and can impact the family, including in the forms of parenting stress and mental health problems (see Yorke et al., 2018, for a review).

Compared to caregivers of neurotypical children and children with other developmental disabilities (e.g., Baker-Ericzén et al., 2005; Hayes & Watson, 2013), caregivers of children with ASD experience increased stress and decreased parenting self-efficacy (Kuhn & Carter, 2006). Importantly, parent stress influences the effectiveness of interventions (Baker-Ericzén et al., 2005). In a study by Krakovich et al. (2016), child behavior accounted for the highest amount of variance of parent stress as compared to other factors, including child intelligence quotient, ASD severity, and level of adaptive skills. Moreover, Worcester et al. (2008) reported that the stress from child behaviors spills over to siblings and extended family members. Parent and family stress is also partially explained by ecological factors. For example, caregivers who live in higher socioeconomic areas may have lower stress levels than caregivers who live in urban areas or in rural areas where support services are not readily available (Bromley et al., 2004; Little et al., 2018b).

Not surprising then, in a survey of parent perceptions of the most effective intervention for child outcomes, in-home behavior supports were identified (Ruble & McGrew, 2007). Caregivers have also reported that interventions in which they have active participation are valuable for themselves and beneficial for their children (Stahmer et al., 2017). Additionally, other studies have found that when caregivers participate in in-home services supplemental to clinic-based services, outcomes are improved (Timmer et al., 2010; Zwaigenbaum et al., 2015).

Access to services is important because parent-mediated intervention approaches demonstrate significant improvement in child developmental skills and other meaningful outcomes (Bearss et al., 2015; Sourander et al., 2016). For child behavior, parent-mediated interventions, in general, are an effective vehicle of change including reduction in parent stress (e.g., Barkley, 1997; Kuravackel et al., 2018; Sourander et al., 2016; Zisser & Eyberg, 2010). For caregivers of children with ASD in particular, parent training has historically been applied as a means for teaching caregivers not only how to address behavior but also support skill development related to social interaction and language acquisition (Green et al., 2010; Kasari et al., 2010; Koegal et al., 1982; Landa, 2018). There are several parent-mediated interventions for behavior available, such as Stepping Stones Triple P—Positive Parenting Program (SSTP; Sanders et al., 2003), Autism Spectrum Conditions—Enhancing Nurture and Development (ASCEND; Pillay et al., 2011), and the Parent Training for Disruptive Behavior Manual developed by Research Units on Behavioral Intervention (RUBI; Bearss et al., 2018b). Data indicate all are effective for improving child behavior and different combinations of other outcomes, including adaptive skill gains (Bearss et al., 2018b), social relationships (Kasperzack et al., 2020), and parenting skills (Pillay et al., 2011). Additionally, Parent-Child Interaction Therapy (PCIT; Eyberg & Funderburk, 2011) showed positive outcomes in parent-child communication and child behaviors in young children (ages 3–12 years) with autism (mean age = 7.15 years old; Allen et al., 2023). While these are notable interventions for effectiveness, none target all three areas of potential outcomes of a parent-mediated intervention for child behavior such as parent competency, stress, and child behavior.

Despite the success of parent-mediated approaches, service access remains a significant barrier for many families (Malik-Soni et al., 2022). According to a number of studies (e.g., Kogan et al., 2008; Resch et al., 2010; Snell-Rood et al., 2020), caregivers have reported frustration with attempting to navigate service systems, which typically only include services limited to outpatient therapy sessions and medication management (Brookman-Frazee et al., 2012). And often behavioral health services are difficult to access because of limited proximity and lack of knowledgeable and experienced service providers (Malik-Soni et al., 2022; Murphy & Ruble, 2012; Scarpa et al., 2020), especially behavior support services from qualified clinicians (Brookman-Frazee et al., 2012; Hodgetts et al., 2015). Other limiting factors include geographic location (e.g., living in a rural area with limited service providers), insurance coverage, family income, and lack of support systems (Nadler et al., 2021). Further, those from culturally diverse backgrounds experience even more barriers to service provision than White families, due in part to lack of providers in culturally diverse areas (McBain et al., 2020), poor elicitation of developmental concerns by healthcare providers from African American and Latino caregivers (Guerrero et al., 2011), and lack of education around ASD traits across cultural groups (Blacher et al., 2019).

For issues of access to trained professionals, telehealth (TH) technology is one viable solution. Not only does TH offer a way to extend services to families in rural areas, it also offers access to more experienced clinicians in ASD. Although the definition of TH interventions spans broadly (Sood et al., 2007), TH involves providing healthcare services, including psychological services, using technology. Although Sood and colleagues explained that defining TH can be complex, the modalities typically associated with TH include: (1) live video or synchronous services, when a provider and client/patient interact in real time using audiovisual telecommunications technology; (2) store-and-forward or asynchronous services, when a practitioner uses secure electronic communications systems to render services to a client/patient that are not in real time; (3) remote patient monitoring, when a provider collects personal data from a client/patient via electronic communication technologies for the purpose of care and support across geographic locations; and (4) mobile health, when a client/patient uses a mobile communication device for health education and support.

One type of TH intervention, asynchronous TH, has gained popularity in the fields of nursing (Spadaro & Hunker, 2016), education (Allaire, 2015), and mental health (Myers & Roth, 2016; Neely et al., 2020). Asynchronous TH refers to a patient and provider communicating with each other at different points in time, rather than in real-time. An advantage of asynchronous TH is accessibility. Caregivers from urban, metropolitan, and rural areas would not be required to alter their busy schedules which often do not permit them to commit to meeting at specific times each week. Asynchronous TH also creates more accessible services for caregivers as there is no need to arrange for transportation to and from a clinic or another site to receive intervention services. Despite these potential advantages, there is no research support for asynchronous TH intervention for ASD, let alone one that targets parent-mediated approaches for addressing child behavior.

The intervention tested in this study, C-HOPE, was developed with the intent to be modified for delivery across a range of modalities, to be accessible to families regardless of geographical location, while maintaining fidelity of intervention implementation and individuality of service delivery. C-HOPE is based conceptually on the Collaborative Model for Promoting Competence and Success (COMPASS; Ruble et al., 2012). COMPASS assumes that competence results from the transaction between the individual and environment. This transaction is considered through an assessment (i.e., COMPASS profile; Ruble et al., 2012) of the child’s personal and environmental supports (protective factors) and personal and environmental challenges (risk factors), as well as the balance between the two. COMPASS is developed specifically for ASD, and it has been tested as a parent-teacher consultation intervention in two randomized controlled trials (RCT; Ruble et al., 2010, 2013) for young children and one RCT for high school age individuals (Ruble et al., 2018) and when implemented by community-trained autism consultants (Ruble et al., 2023).

C-HOPE retains key structural and procedural elements of the original COMPASS model, including the collaborative goal-setting process, the use of individualized behavior plans derived from the COMPASS profile, and follow-up coaching to support implementation fidelity. Whereas COMPASS was originally developed as a teacher consultation model targeting educational goals, C-HOPE adopts these same principles for a parent-mediated format that emphasizes managing problem behaviors and reducing parenting stress within the home environment. Thus, the primary change from COMPASS to C-HOPE involved the shift in recipient (from teachers to parents) and delivery format (from school-based consultation to therapeutic parent individual and group sessions), while maintaining the same conceptual and procedural foundation.

Using a randomized wait list control pre-post design, C-HOPE was tested with caregivers of young children with ASD and results revealed significant pre-post changes in the three main outcome areas of child behavior, parenting stress, and parenting sense of competency, with effect sizes ranging from 0.54 to 0.71 (Kuravackel et al., 2018). Further, analyses revealed no significant differences based on modality of delivery (FF or traditional TH format delivered synchronously) for child behavior, parent stress, and parenting competency. Additionally, parent satisfaction and therapist fidelity were high and similar for both the FF and TH conditions. In the Kuravackel et al. (2018) study, “child behavior” referred specifically to reductions in parent-reported disruptive and problem behaviors, as measured by the Eyberg Child Behavior Inventory (ECBI), rather than changes in core ASD symptoms such as social communication or restricted and repetitive behaviors.

Although the present study was primarily quantitative, caregiver feedback was also collected to provide insight into families’ experiences with the hybrid asynchronous format. Understanding caregiver perceptions of engagement, satisfaction, and feasibility is an important component of evaluating the acceptability of hybrid telehealth interventions (e.g., Bearss et al., 2018a; Su et al., 2023). These comments are presented descriptively to illustrate parent perspectives rather than as formal qualitative findings.

The purpose of this study was to examine the impact of a parent-mediated hybrid TH intervention for ASD on the three major outcomes assessed previously—parent competency, child behavior, and parent stress. The primary aim of this study was to compare pre- and post-outcomes of C-HOPE delivered in a hybrid asynchronous/synchronous format. In this version, families interacted with a therapist facilitator asynchronously through Canvas discussion boards and received written feedback and individualized coaching, supplemented by scheduled individual teleconferencing sessions.

A secondary, descriptive aim was to compare these findings with previously published synchronous and face-to-face C-HOPE data (Kuravackel et al., 2018) to contextualize results rather than to conduct a direct statistical comparison. Because the present hybrid design retained the same number and length of sessions as the original C-HOPE study (Kuravackel et al., 2018), comparisons are descriptive rather than inferential. Furthermore, the concept of the therapeutic alliance was evaluated in the study to determine if the format of the intervention interfered with the therapeutic alliance. Extensive research consistently highlights the therapeutic alliance, or the strength of the client–therapist relationship, as one of the most reliable predictors of successful outcomes in therapy and intervention (Flückiger et al., 2018; McGrew et al., 2016; Safran & Muran, 2000). the quality, maintenance, and repair of the therapeutic alliance are foundational to effective psychological intervention. Effective Interventions involve not only building the alliance but attending to its strains, repairing ruptures, and thereby transforming the alliance into a deeper, more collaborative, and growth-oriented relational space (Safran & Muran, 2000). McGrew et al. (2016) emphasize that the success of autism interventions depends heavily on how well the clinician engages with the family, adapts the intervention to their context, and implements it with fidelity, beyond just selecting an evidence-based intervention.

The present study had the following objectives:

(1) To compare pre- and post-intervention outcomes for child behavior, parent stress, and parenting sense of competence among caregivers who completed the hybrid version of C-HOPE;
(2) To descriptively compare these outcomes with previously published results from the synchronous face-to-face and telehealth implementations of C-HOPE (Kuravackel et al., 2018) to contextualize findings across modalities;
(3) To examine therapeutic alliance within the hybrid delivery format to evaluate whether a positive therapeutic relationship could be maintained when combining asynchronous group discussion boards with synchronous individual coaching.

It was hypothesized that: (a) caregivers would report significant pre- to post-improvements in child behavior and parenting stress, with increased parenting sense of competence; (b) outcomes for the hybrid delivery would be comparable in direction and magnitude to those observed in prior synchronous and face-to-face C-HOPE iterations; and (c) therapeutic alliance ratings would remain in the good-to-excellent range, indicating that engagement and collaboration were preserved across modalities.

## 2. Method

### 2.1. Intervention

C-HOPE is a parent-mediated intervention designed to address child behavior, parent stress, and parenting sense of competence through the use of evidence-based practices (EBPs). Caregivers are trained in specific EBPs, including positive reinforcement, behavior shaping, and relaxation techniques, which they implement with their children in everyday settings. The selection of EBPs is tailored to each family’s unique challenges, with the therapist and parent collaborating to identify the most effective strategies for the child’s specific behaviors and the family’s goals. The hybrid asynchronous format evaluated in the present study maintained the same structure and dosage as the original synchronous C-HOPE delivery described by Kuravackel et al. (2018), which included eight sessions (four 2 h group and four 1 h individual meetings) delivered either face-to-face or via synchronous telehealth. In both versions, facilitators provided direct coaching to caregivers, and caregivers completed between-session practice activities that required approximately one to two hours per week.

Functional Behavior Assessments (FBAs) were not formally conducted in this study; however, participants were coached to observe and identify patterns in their child’s behavior, including antecedents, triggers, and consequences. This informal analysis allowed caregivers to adjust reinforcement and intervention strategies in real time based on their observations.

### 2.2. Participants

Eligible participants were primary caregivers who had a child (1) with ASD; (2) with behaviors that were mismatched to parent expectations, excluding extreme physical aggression and self-harm; and (3) between the ages of 3–12 years. Children’s ASD diagnoses were previously established through clinical or educational evaluation and verified through parent reports or documentation provided during recruitment. For eligibility confirmation, participants completed one of two screening measures for autism: the Modified Checklist for Autism in Toddlers, Revised with Follow-Up (M-CHAT-R/F; Robins et al., 2009) or the Social Communication Questionnaire (SCQ; Rutter et al., 2001), depending on age. Both instruments are widely used in research and clinical settings as screening measures for ASD (e.g., M-CHAT-R/F: sensitivity = 0.83, specificity = 0.94; Aishworiya et al., 2023; SCQ: AUC ≈ 0.885; Chesnut et al., 2017). Of the families who expressed interest and met eligibility criteria, 15 enrolled in the program. Ten families, however, completed the intervention and post-intervention assessments, and five did not receive any intervention. No participants withdrew because of dissatisfaction or adverse events. The number of families screened prior to enrollment was not recorded. Caregivers who completed and those who did not complete the intervention were generally similar in demographic characteristics (e.g., child age, gender, and family income) and service involvement, based on available data. However, formal analyses of these differences could not be conducted because the original dataset is no longer accessible. The first author’s university’s Office of Research Integrity reviewed and provided approval for this study and its materials and procedures.

Participants had to have access to a telephone and Internet connection with either a computer, tablet, or smartphone, and attend training for using the online platform Canvas Free for Teachers (Instructure, 2018) prior to beginning treatment. Families were ineligible if they: (1) had another child already in the study or (2) were not comfortable with the assessments or intervention conducted in English. Families who were deemed ineligible to participate were referred to ASD resources and/or services in their local communities.

Participants were recruited through organizations supporting individuals with ASD and their families (e.g., local Autism Society of America chapters, other support groups, listservs, and Facebook groups), as well as three university/hospital clinics serving individuals with ASD and their families. Fifteen families enrolled in the study. A small number of interested participants (*n* = 5) were unable to participate due to limited Internet access or time to participate in the study. No families were excluded for severe behavior problems. As reflected in Table 1, the sample was predominantly higher income, possibly reflecting differential access to the required technology; this limitation is discussed later.

Participants were screened for eligibility over the telephone during the recruitment process. Informed consent was obtained electronically. Participants were emailed an IRB-approved consent form and then provided consent by selecting “yes” on the first page of the Qualtrics baseline survey, which contained the same consent language. Participants were randomly assigned, using a random number generator, to one of two treatment groups, which began two weeks apart to accommodate scheduling and facilitator workload. Participants were informed of group assignment one week before the first group was asked to complete baseline evaluations, and the facilitator did an additional “check-in” with participants assigned to the second group one week before they were asked to complete baseline evaluations.

Fifteen participants from across the states of Virginia, Ohio, and Kentucky were recruited. Participant demographic characteristics are presented in Table 1, which also includes descriptive demographic comparisons between the current hybrid C-HOPE sample and the original C-HOPE sample (Kuravackel et al., 2018) to contextualize findings. The mean age of the children in the current study was 7.9 years; 73% were male. Parent participants were 100% female per self-report. The majority, 73%, of the children were White and 27% were Black. For household income, about 7% earned less than $10,000, 7% earned $25,000–49,999, 47% earned $50,000–99,999, and 40% earned $100,000 or more. Approximately 26% of participants lived in rural counties as defined by the U.S. Census Bureau (Ratcliffe et al., 2016). Finally, approximately 53% of participants’ children were receiving some other kind of behavioral or mental healthcare service, such as applied behavior analysis or individual therapy. The remaining 47% were not receiving other services (see Table 1).

After recruitment, participants were randomly assigned to either a group that started immediately or one that started two weeks later. The first group consisted of eight caregivers; the second group consisted of seven caregivers. Participant demographics were generally comparable to those reported in the original synchronous C-HOPE study (Kuravackel et al., 2018), with similar distributions in child age, gender, race, and family socioeconomic status. Table 2 shows the sessions’ duration, format, and goals. The individual sessions occurred via telephone, and the group sessions were completed asynchronously with Canvas. All data were collected using Qualtrics.

## 3. Measures

### 3.1. Autism Screeners

As mentioned, two screeners were used to verify eligibility: the (M-CHAT-R/F (Robins et al., 2009) and the SCQ (Rutter et al., 2001). The M-CHAT-R/F is a 20-item parent-report screening tool for identifying risk for autism in young children aged 16–30 months and demonstrates pooled sensitivity of 0.83 and specificity of 0.94 (Aishworiya et al., 2023). The SCQ is a 40-item caregiver questionnaire designed to screen for autism characteristics in individuals aged 4 years and older, with meta-analytic area under the ROC curve of 0.885 (Chesnut et al., 2017). Both are validated screening instruments and were used in this study to further validate caregiver-reported autism diagnoses for eligibility purposes. Screeners were administered by facilitator via telephone call. All remaining measures described below were administered via Qualtrics.

### 3.2. Child Behavior

The Eyberg Child Behavior Inventory (ECBI; Eyberg & Pincus, 1999) is a 36-item parent-report measure of conduct-problem behavior in children between the ages of 2 and 16 years. The 36 items are rated on two scales: (a) The Intensity Scale asks for a frequency of occurrence rating for each behavior item and (b) The Problem Scale asks the parent to identify the problem behaviors (yes/no responses), with the sum of “yes” responses yielding the problem score. Only the total score, or Problem Scale score, was used in data analysis for the current study. Reliability of the ECBI was supported in the current sample, with an internal consistency of α = 0.89, demonstrating good reliability. Construct validity has been established in previous studies with diverse populations (Abrahamse et al., 2015; Gross et al., 2007; Rhee & Rhee, 2015), confirming its suitability for assessing behaviors in children with ASD. The ECBI was administered at baseline and again following program completion to assess changes in child problem behaviors.

### 3.3. Parent Stress

The Parenting Stress Index—Fourth Edition (PSI-4; Abidin, 2012) is a standardized parent-report questionnaire designed for caregivers of children ranging in age from 1 month to 12 years. Items are scored on a 5-point Likert-type scale (1 = “Strongly disagree”, 5 = “Strongly agree”). The subscales of the PSI-4 are grouped into two domains: the Parent Domain and the Child Domain that are also used for a total stress score. The Child Domain measures caregivers’ perception of the demands of caring for the child. The Parent Domain measures caregivers’ reaction to the demands of caring for the child. The total Stress scale was used for this study. The PSI-4 has high internal consistency (*α* ≥ 0.96) and test-retest reliability, with coefficients ranging from 0.65 to 0.96 (Abidin, 2012). In the current study, internal consistency for the PSI-4 was strong, with *α* = 0.90. These results align with previous studies that have validated the PSI-4 in ASD parent samples (Lee et al., 2016; Silva & Schalock, 2012; Touchèque et al., 2016). The PSI-4 was administered at baseline and post-intervention to measure changes in overall parenting stress across the course of the program.

### 3.4. Parenting Sense of Competence

The Being a Parent Scale (BPS; Johnston & Mash, 1989) is a 16-item questionnaire measuring caregivers’ views of their competence as caregivers on dimensions of their satisfaction with their parenting role (reflecting the extent of frustration, anxiety, and motivation) and feelings of their efficacy as a parent (reflecting competence, problem solving ability, and capability in parenting role). Items are scored on a six-point Likert scale (Strongly agree to Strongly disagree), with higher total scores on the BPS indicating high parenting sense of competence. In this study, internal consistency for the BPS was good (*α* = 0.82), consistent with previous research. Johnston and Mash (1989) reported high levels of internal consistency for the Total Score (*α* = 0.79), Satisfaction factor (*α* = 0.75), and Efficacy factor (*α* = 0.76). These findings provide evidence for both the reliability and construct validity of the BPS for assessing parenting sense of competence. The BPS was administered at baseline and post-intervention to assess changes in caregivers’ self-perceived competence over time.

### 3.5. Therapist, Client, and Relationship Variables

Therapist fidelity and parent satisfaction were assessed with scales developed specifically for C-HOPE. Implementation fidelity of C-HOPE was measured as a percentage of whether the facilitator carried out critical elements of each session, according to the parent report. For example, caregivers endorsed “yes” or “no” regarding whether specific topics were covered, whether the session incorporated appropriate materials and ideas, etc. A sample fidelity checklist is included in Appendix A. Internal consistency for the fidelity checklists was good (*α* = 0.88), supporting their reliability. Satisfaction was measured based on parent perceptions of the session, according to a Likert scale ranging from 1 “Extremely Dissatisfied” to 4 “Extremely Satisfied.” Examples of questions were “how much did [the parent] feel involved in the session,” “…feel listened to,” etc. Internal consistency for the satisfaction questionnaires was good (*α* = 0.81), supporting their reliability.

The Outcome Rating Scale (ORS; Miller & Duncan, 2000) was used to assess parent well-being from session to session. Participants were given Qualtrics links to complete the ORS at the beginning of each session. The ORS is a measure of client well-being intended for progress monitoring. The ORS comprises four items, which are each rated by the client using a 10 cm line visual analog scale. Clients are instructed to place a mark on each line, with low estimates toward the right of the line and high estimates toward the left. The ORS has a maximum score of 40, with scores 25 and lower indicating need for a helping relationship. Internal consistency for the ORS was excellent (*α* = 0.92), supporting their reliability. The ORS was administered at the beginning of each session to monitor ongoing caregiver well-being.

The Session Rating Scale Version 3 (SRS; Johnson et al., 2000) and Group Session Rating Scale (GSRS; Duncan & Miller, 2007) were used as measures of therapeutic alliance, which refers to the collaborative and trusting relationship between a therapist and client. Therapeutic alliance plays a key role in the success of psychological interventions, and it involves three essential elements: (1) agreement on goals, (2) agreement on tasks, and (3) the development of a personal bond between the therapist and the client (Bordin, 1979). Research consistently shows that a strong therapeutic alliance is linked to better outcomes across different types of interventions. More recent studies, such as Flückiger et al. (2018), highlight therapeutic alliance as a dynamic process involving mutual collaboration, trust, and emotional connection, further supporting its role as a predictor of successful treatment outcomes. Participants were given Qualtrics links to complete these measures following each session (i.e., SRS following individual sessions, GSRS following group sessions). The SRS and GSRS are measures of alliance which encourage regular engagement between therapist and client regarding their relationship. Similarly to the ORS, the SRS and GSRS each comprise four items, rated using the same visual analog scale and scoring system. Scores from 0–34 indicate poor alliance, 35–38 indicate fair alliance, and 39–40 indicate good alliance. Internal consistency for both the SRS (*α* = 0.94) and the GSRS (*α* = 0.92) was excellent, supporting their reliability. The SRS and GSRS were administered at the end of individual and group sessions, respectively, to evaluate therapeutic alliance.

## 4. Procedure

C-HOPE is a manualized intervention. Through the iterative approach taken to developing and refining C-HOPE across its different modalities and across time and geographical location, the researchers have developed a comprehensive treatment manual guiding the facilitator through each session, complete with tips for carrying out sessions via TH versus FF. Accompanying the treatment manual is a parent workbook, complete with all session handouts and forms, copies of PowerPoint presentations for group sessions, and additional resources for families.

The aim of this study was to test the efficacy of an asynchronous adaptation of C-HOPE, which utilized pre-recorded group sessions. The study was a 10-week long, pre-post design using TH technology. Although individual sessions were conducted via telephone (similar to the pilot study by Kuravackel et al., 2018), group sessions were pre-recorded using an iPad mounted on a tripod, with a television monitor showing the PowerPoint presentations. The facilitator presented the group material just as it had been presented in previous iterations of C-HOPE, incorporating prompts for participants to post in an online discussion board when relevant topics were introduced.

The pre-recorded group session videos were edited to include visual reminders for participants to complete forms and participate in discussion board topics. These videos were then uploaded to Canvas, a secure learning management system incorporating cloud-native software, often utilized for online classes in public and higher education. Canvas housed all session materials, including discussion boards and handouts, accessible to participants at their convenience. Participants were provided instructions on creating a free Canvas account and navigating the platform.

Targeted behaviors were clearly operationalized through structured collaboration between the facilitator and the parent. For example, “tantrum” was defined as “throwing objects, hitting, or yelling for more than 30 s.” This process ensured that data collection was accurate and reliable. Although interobserver agreement (IOA) was not formally used in this study, caregivers tracked behavior changes using detailed tracking forms, and the facilitator provided support in interpreting the data. Participants were encouraged to watch the group sessions and engage with the discussion board asynchronously, allowing flexibility regarding when and where they participated. They were instructed to participate during a time with limited distractions and in a private location to maximize focus and engagement with the material. While there was no set time for viewing sessions, a suggested deadline for each session (i.e., by Thursday evening) was provided to encourage timely engagement on the discussion board before each session week concluded.

All group interaction occurred asynchronously in written form within the Canvas discussion boards. The facilitator initiated each topic with written prompts, monitored participation daily, and responded to parent posts to sustain dialogue and provide coaching, thereby remaining actively involved throughout the discussion period. The asynchronous group discussions were facilitated by a doctoral psychology intern completing a predoctoral internship, under licensed supervision. The facilitator initiated weekly discussion prompts, monitored parent participation, and provided individualized written feedback within the Canvas platform to guide reflection and application of strategies. The same facilitator also conducted individual teleconferencing sessions with each family to provide personalized coaching and support.

Facilitator fidelity was measured using session-specific fidelity checklists, which ensured adherence to the C-HOPE manual and the core components of each session. The fidelity checklists, completed by participants following each session, covered key session elements such as presenting session goals, reviewing relevant materials, guiding discussion, and encouraging parent engagement. Participants also provided feedback on whether the critical components were addressed during sessions. An example of a fidelity checklist used for Group Session 1 is included in Appendix A. Internal consistency for the fidelity checklists was strong (α = 0.88), suggesting that the intervention was delivered as intended across all sessions.

The C-HOPE manual guided the content and pacing of each session, ensuring consistency with the pilot study. Ongoing discussion was encouraged throughout each week to promote continued engagement with the material and with other participants. The study’s structure and session topics are outlined in Table 2.

## 5. Data Analysis Plan

For the first research question of whether asynchronous C-HOPE results in increased parent competency (BPS), decreased parent stress (PSI-4), and reduced child behavior (ECBI), paired sample *t*-tests were conducted to examine mean differences on pre- and post-intervention variables. Paired sample *t*-tests were also used to examine parent outcomes (ORS) and therapeutic alliance (SRS/GSRS). Facilitator fidelity and parent satisfaction were analyzed using percentages and means. Groups were analyzed and compared at baseline and final time points to ensure no group differences existed. Statistical Package for the Social Sciences version 24.0 (SPSS 24.0) was used. Due to the small sample and attrition, analyses were conducted using a completer-only approach (n = 10). Intention-to-treat analyses were not feasible because no post-intervention data were available for the five non-completing participants.

## 6. Results

Main Outcomes: Child Behavior, Parent Stress, and Parenting Sense of Competency.

Analysis of changes in parenting sense of competence (BPS) revealed no significant difference in pre-intervention and post-intervention scores (*p* = 0.30, Cohen’s *d* = 0.27). In contrast, a significant difference was revealed for child behavior, with the ECBI showing a decrease in behavior from pre- to post-intervention (*p* = 0.01, Cohen’s *d* = 0.73). A significant difference was also observed for parent stress, as measured by the PSI-4 (*p* = 0.03, Cohen’s *d* = 0.50). Parent well-being scores, as measured by the ORS, improved significantly from the first session to the eighth session (*p* = 0.00, Cohen’s *d* = 0.59). These results suggest that participation in asynchronous C-HOPE is associated with notable improvements in both parent and child outcomes (see Table 3 for all main outcome *t*-test results).

In addition to these primary outcomes, session-level well-being was also monitored using the Outcome Rating Scale (ORS; Miller & Duncan, 2000). Although the ORS was not one of the study’s primary outcome measures, it was used to track caregiver well-being across sessions. Results indicated a statistically significant improvement in ORS scores from the first to the final session, *t*(9) = −3.64, *p* < 0.01, with a medium-to-large effect size (Cohen’s *d* = 0.59). The higher degrees of freedom for this analysis reflect that the ORS was administered at the beginning of each session, providing more data points than the pre-post outcome measures. These findings suggest that caregivers experienced incremental improvements in well-being throughout the course of the intervention.

Caregivers reported observing positive changes in their child’s behavior after participating in asynchronous C-HOPE and applying the skills they learned. While a formal FBA was not conducted, caregivers worked collaboratively with the facilitator to operationally define target behaviors, as described in Section 4. Behavior plans were developed and refined throughout the intervention based on these operational definitions, allowing caregivers to apply positive reinforcement and other strategies in everyday situations. Anecdotally, many caregivers reported following through on the proposed behavior plans developed during Individual Session 2 and modifying them throughout the remaining sessions. See Table 4 for additional parent-reported examples of behavior changes.

Although the sample size precluded formal correlational analyses, we explored potential associations among demographic variables, participation, and outcomes descriptively. Course utilization data were collected at the macro level (i.e., aggregated activity within the Canvas platform) rather than the individual participant level. Therefore, engagement metrics such as time spent on Canvas, number of discussion posts, and video views could not be linked to specific participants or analyzed in relation to outcome improvements. In addition to the standardized outcome measures, caregiver comments drawn from the asynchronous Canvas discussion boards were used to provide illustrative examples of families’ engagement and perspectives during the program. These quotes represent naturally occurring reflections shared as part of the intervention rather than data collected through a separate qualitative process. They were used descriptively to contextualize caregiver experiences and were not analyzed thematically or through formal qualitative methods.

## 7. Parent Feedback for Contextual Insight

In addition to quantitative data, caregivers provided rich feedback regarding their experiences with the intervention. Although no formal qualitative analysis was conducted, selected quotes are included here to illustrate the impact of the program. For example, one parent shared, “My child’s behavior definitely plays a role in my stress level. It often determines where I go and who I am around when my child is with me.” Another parent shared similar sentiments: “Even with behavioral strategies, I feel like I don’t try hard enough. … It starts to make me feel paralyzed sometimes.” These insights help contextualize the interconnectedness of child behavior, parent stress, and parental self-efficacy, which is supported by the quantitative findings.

Caregivers also reflected on specific components of the intervention, such as their use of relaxation techniques and identifying replacement behaviors. For example, one participant described using emotional grounding techniques to manage stress in a real-life situation: “I definitely use more of the soothing grounding, especially when I am in the car going from place to place with fighting and screaming kids in the back seat.” See Table 4 for additional examples.

Although caregivers frequently described the discussion board as a helpful aspect of the program, the present design did not allow for direct comparison of outcomes with and without this feature. Accordingly, while the discussion board may have enhanced engagement or perceived support among participants, its role as an active ingredient in the intervention cannot be determined. Future studies could systematically examine the contribution of discussion board participation to treatment outcomes.

## 8. Secondary Outcomes: Treatment Fidelity and Caregiver Satisfaction

Facilitator treatment adherence, as measured by session-specific fidelity checklists, ranged from 85.50% to 100.00% (*M* = 95.37, *SD* = 4.52). Parent-reported satisfaction with sessions, as measured by satisfaction questionnaires, was also high (*M* = 3.60, *SD* = 0.20). Despite high satisfaction, several participants commented that the time commitment required for sessions and data collection outside of sessions was challenging, particularly in an asynchronous format where there was no live interaction with other caregivers. Finally, therapeutic alliance was fair according to the SRS (*M* = 36.80, *SD* = 2.80, range = 26.30–40.00) and GSRS (*M* = 36.80, *SD* = 2.82, range = 26.30–40.00). Alliance across sessions did not improve significantly from the first session (*M* = 32.35, *SD* = 6.54) to the final session (*M* = 35.25, *SD* = 6.59).

Course utilization data were collected at the macro level (i.e., aggregated activity within the Canvas platform) rather than the individual participant level. Therefore, engagement metrics such as time spent on Canvas, number of discussion posts, and video views could not be linked to specific participants or analyzed in relation to outcome improvements.

## 9. Course Utilization Data

Canvas, the cloud-native learning management system used to host the group session videos and discussion boards, as well as course files/handouts, collected data regarding usage of the discussion board and other course pages by the participants. Total time spent engaged in the Canvas platform ranged from 320 min, or five hours and 20 min, to 683 min, or 11 h and 23 min (*M* = 516.00, *SD* = 133.29). The time spent engaged on Canvas did not include group session viewing times, as the videos were housed privately on Vimeo. Total page views (i.e., opening particular discussion board and course material links, including accessing the group session Vimeo links) ranged from 128 to 303 (*M* = 231.91, *SD* = 60.92). Across all four group sessions, 41 different discussion topic prompts were posted by the facilitator (i.e., 9 to 11 topics per session). Participants were encouraged to participate in as many discussion board topics as they felt comfortable, but no minimum number was set as an expectation. Discussion board posts per participant across the four group sessions ranged from 12 to 41 (*M* = 29.36, *SD* = 10.08). Overall, it appeared that participants were engaged and interacting with the Canvas course material and completing group sessions as expected. Table 4 above provides examples of discussion board prompts and participant responses per session.

In addition to the quotes from caregivers drawn from the discussion boards, participants were also encouraged to provide feedback following each group and individual session. Some comments made by caregivers following sessions included, “learning new concepts that my child may have or need to work on is helpful in understanding [his] overall reactions to things,” “seeing comments from the other caregivers [was a strength],” and “I appreciated … this last session where we were asked to make a plan for our own well-being (this is not something I would do if not asked to do it).” Caregivers also provided constructive feedback. Specifically, the biggest challenge noted most often by participants was the time commitment of one to two hours per week for sessions and additional time dedicated to implementing plans and tracking data outside of sessions. Participants sometimes noted that they would not have minded the two-hour time commitment for group sessions as much had they been able to interact with the other caregivers in person as opposed to completing the sessions on the computer independently. Given the small sample size, formal analyses of potential correlates were not conducted. However, descriptive implementation data indicated variability in families’ participation in discussion boards and use of module materials.

## 10. Comparison to Original C-HOPE

As noted previously, the original C-HOPE study by Kuravackel et al. (2018) tested the intervention delivered synchronously via telehealth and face-to-face modalities. Although direct statistical comparison between studies was not possible, demographic characteristics, session structure, and intervention duration were similar across formats. In that study, improvements were reported for child problem behavior (*d* = 0.73), parent stress (*d* = 0.54), and parenting sense of competence (*d* = 0.32). In the current asynchronous hybrid format, we observed a similar pattern of results, with large and medium effect sizes for child behavior (*d* = 0.73) and parent stress (*d* = 0.50), respectively, and a smaller, nonsignificant change for parenting sense of competence (*d* = 0.27). These comparable magnitudes of change suggest that the hybrid asynchronous/synchronous approach can achieve outcomes consistent with those found in synchronous or face-to-face delivery formats, supporting the flexibility and scalability of the C-HOPE model across telehealth modalities.

## 11. Discussion

The present study examined outcomes of a hybrid asynchronous adaptation of C-HOPE, a parent-mediated behavior intervention based on the COMPASS framework (Ruble et al., 2012). The primary objective was to determine whether the asynchronous group format, supplemented by individualized telehealth sessions, could yield outcomes comparable to the original synchronous delivery (Kuravackel et al., 2018). C-HOPE targets three interrelated domains that have been shown to influence one another in behavioral parent training (BPT) models—child challenging behavior, caregiver stress, and parenting sense of competence (Bloomquist, 2017; Hastings, 2002). Within this framework, changes in child behavior can reduce parent stress, and reduced stress can, in turn, enhance caregivers’ sense of efficacy. However, the current investigation focuses primarily on evaluating whether the hybrid asynchronous delivery model can maintain intervention fidelity and caregiver outcomes consistent with the synchronous version, rather than testing directional relationships among these constructs.

This study is unique to others that have been published examining parent-mediated behavior intervention programs in that it considers all three major factors of child behavior, parent stress, and parenting sense of competence—other programs typically target only one or two of those three key areas. Additionally, it was crucial to evaluate factors of treatment fidelity and caregiver satisfaction due to these being common factors on treatment outcomes, including the therapist, the client, and the relationship variables (McGrew et al., 2016) and whether the asynchronous delivery interfered with therapeutic alliance. One of the most novel features of this study is the utilization of a web-based asynchronous discussion board for group sessions that allowed caregivers to interact with one another and gather input and feedback. The web-based feature is an important use of technology for families from various diverse groups who have been shown to have difficulty accessing appropriate clinical services, including those living in rural locations (Nadler et al., 2021; Thomas et al., 2007).

The results of this study suggest that asynchronous C-HOPE had a meaningful impact on both parent and child outcomes. Caregivers reported a significant decrease in child behaviors, which highlights the potential for asynchronous parent-mediated interventions such as C-HOPE to effect behavior change. Although a formal FBA was not conducted, the operationalization of child behaviors and the collaborative development of behavior plans likely contributed to these improvements. These findings are consistent with a recent meta-analysis suggesting that parent-mediated interventions for children with ASD are associated with positive behavior change (Conrad et al., 2021). Caregivers participated in the sessions with strong behavioral tracking data, questions about plan implementation, and feedback regarding how they saw the plan working for their family and their child’s life. Further, the opportunity for caregivers to interact asynchronously on the group discussion board, ask questions of each other, and share success in their current plans and with regard to techniques that have worked for them in the past, is unique and fills a gap in the research. Although many existing intervention programs feature a group component, the main focus tends to be on behavioral psychoeducation during group sessions, with some programs (e.g., Sofronoff et al., 2004; Stuttard et al., 2016) allowing for discussion on these particular topics. C-HOPE is unique in that it encourages group discussion regarding a range of psychoeducational topics presented (i.e., ASD traits, behavior, behavioral management, caregiver stress, and ways to cope). Questions are posed to encourage participants to think about the material, apply it to their lives, and share with others the connections they made regarding their own children and their behaviors.

The significant reduction in parent stress post-intervention, with a medium effect size, is especially meaningful. This is consistent with research indicating that interventions incorporating psychotherapeutic components for caregivers, such as cognitive behavioral therapy and mindfulness skills, can help reduce stress and improve well-being for caregivers of children with developmental disabilities (Li et al., 2024). Unlike many behavior-focused programs that do not address parent well-being, C-HOPE intentionally addresses this gap through teaching caregivers various therapeutic techniques for stress management, encouraging them to think about their stress levels and the impact it has on their lives in general, and leading them through creating their own stress prevention and wellness plans. The focus on parent stress in both group and individual sessions may explain the positive outcomes in this area.

The original C-HOPE and the current hybrid version followed the same eight-session structure with comparable family demographics and service involvement, but the present design replaced live group meetings with asynchronous discussion boards. This consistency supports interpreting the results as preliminary evidence that similar outcomes can be achieved even when group sessions are delivered asynchronously. Unlike the original C-HOPE study (Kuravackel et al., 2018), no change in parent-reported sense of competence was revealed. This may be because of the asynchronous nature of the group discussion board or the small sample size. A study by Kurzrok et al. (2021) revealed that caregivers of children with ASD report higher sense of competence when they are more involved in their children’s interventions and are more satisfied with intervention-related training. Unlike the synchronous intervention described by Kurzrok et al. (2021), in which parents received real-time clinician feedback and interactive guidance, the current hybrid asynchronous model relied primarily on written facilitator feedback within discussion boards. The reduced opportunity for live coaching and immediate reinforcement of strategies may have limited growth in caregiver sense of competence, even as caregivers demonstrated improvements in stress and child behavior outcomes.

Further, higher financial and social burdens are correlated with lower parenting sense of competence (Kurzrok et al., 2021), and burden was not assessed in this study. On the other hand, BPS scores appeared somewhat high at baseline, suggesting that a ceiling effect may have occurred. A ceiling effect occurs when participants’ scores approach the maximum possible value on a measure, leaving little room to detect improvement even if change has occurred. Future research may benefit from using alternative or more sensitive measures of parenting self-efficacy, such as the Parenting Sense of Competence Scale or the Parent Efficacy and Empowerment Scale, to better capture changes among parents with high baseline competence levels. Further, lack of significant improvement in parenting sense of competence does align with findings from some similar studies, as described below.

Research on family interventions for ASD, such as the ECHO Autism family navigation program, has shown that parent competence often remains unchanged unless the intervention includes more direct parental involvement in the therapeutic process or more opportunities for reflective feedback (Mazurek et al., 2025). Although caregivers in the current hybrid asynchronous model were highly engaged in implementing strategies and participating in discussion boards, their involvement occurred primarily through self-directed activities rather than through real-time coaching. This structure provided fewer opportunities for interactive reflection and immediate feedback from a facilitator, which may help explain why caregiver sense of competence did not significantly increase despite consistent engagement. Another possible explanation is that the focus of the current iteration of C-HOPE on asynchronous group discussion and behavior management strategies may not directly target the areas that caregivers associate with their sense of competence, such as measures of active problem-solving and confidence-building (Musetti et al., 2021).

In the original C-HOPE study (Kuravackel et al., 2018), facilitators spent approximately 12 h per family across eight sessions (four 2 h group and four 1 h individual sessions). In the present hybrid format, the facilitator provided roughly six hours of scheduled individual contact and an additional one to two hours per week of asynchronous discussion moderation. Families reported dedicating about one to two hours weekly to reviewing module materials and contributing to discussion boards. These estimates indicate that the hybrid model reduced real-time scheduling demands while maintaining similar overall intervention dosage.

In future iterations of C-HOPE, adding elements that foster increased parental reflection on their growth, such as journaling or more frequent one-on-one check-ins, may help improve their sense of competence. Further, interventions that specifically provide opportunities for caregivers to practice new skills in a real-time, guided format (similar to PCIT) could further support this outcome. These adaptations could help align the C-HOPE framework more closely with the broader needs of caregivers in terms of confidence and efficacy in their parenting roles. Anecdotally, several parent participants commented that participation in C-HOPE increased their confidence in their abilities to do their jobs as caregivers of children with ASD. Perhaps with more iterations of this line of research, incorporating the adaptations discussed here, we will find a significant increase in parenting sense of competence after completion of C-HOPE, specifically within the asynchronous group discussion modality.

Results indicated that caregivers reported being satisfied with the C-HOPE individual and group sessions overall, and they also felt that the facilitator implemented the intervention with fidelity. Additionally, parent well-being scores improved significantly from the first session to the final session, which suggests that caregivers felt better individually, interpersonally, socially, and/or overall, after participating in C-HOPE. Finally, therapeutic alliance was fair (Duncan & Miller, 2007; Johnson et al., 2000), with a mean score of 36.80 out of 40.00 across all sessions, as rated by parent participants. This is important as suggested by ample literature (e.g., Flückiger et al., 2018; McGrew et al., 2016; Safran & Muran, 2000) documenting alliance or a strong therapeutic relationship as one of the best indicators of client outcome in intervention and therapy. Regarding this study, the therapeutic relationship was built through not only interaction with the facilitator on the group session discussion boards, but also on the telephone during individual sessions via attentive and empathic listening, asking of relevant questions, and connecting ideas shared from session to session and across group and individual sessions.

Results of the present study replicated results from the original study (Kuravackel et al., 2018) for treatment adherence and parent satisfaction. Both were rated high. Also replicated was the finding that parent well-being scores increased over the course of C-HOPE. Therapeutic alliance was also consistent with the study by Kuravackel et al. (2018). The consistent results across studies suggest that C-HOPE can be implemented with fidelity, is perceived as socially valid, and produces similar outcomes to parent-reported well-being and therapeutic alliance, across time, geographic location, and researcher/therapist. Further, C-HOPE outcomes were similar regardless of modality (i.e., FF, traditional TH, and asynchronous group discussion board formats).

Interventions that can be delivered successfully using TH and other technologies is particularly relevant today given COVID-19. In light of the increased availability of TH services since the onset of COVID-19, it is important to note that engagement of caregivers in online coaching/support programs can be high when there is strong communication and support as well as opportunity to share personal experiences and beliefs, although overwhelm can occur when there are multiple tasks assigned through the support process (Ogourtsova et al., 2021). However, investigation into outcomes of ASD-specific asynchronous TH interventions is limited (Simacek et al., 2021). Demonstrations of success of asynchronous interventions have been noted in other fields, including dermatology (Muthiah et al., 2023), dentistry (Flores-Hidalgo et al., 2023), and adult behavioral health (Walsh et al., 2023). Recently, more researchers have begun to focus on ASD-specific treatments that include an asynchronous TH component, such as in delivering speech-language therapy (Karrim et al., 2022) and occupational therapy (Little et al., 2023). The current study works to further expand on the limited literature for this particular population and treatment focus.

Further, the course utilization data collected reflects the ability and willingness of caregivers who seek services to follow through with the expectations of clinical interventions delivered asynchronously. Caregivers accessed the group session videos, viewed relevant handouts and links suggested to them, and participated in the discussion boards, oftentimes spending several hours per group session viewing materials, adding their own comments to the discussion board, and responding to the comments of others in the group. These results begin to provide support for the use of asynchronous group interventions for engaging caregivers of children with ASD who have a need for services.

Although there are several strengths of the study, caregivers also noted challenges to the program when asked to provide feedback. Specifically, they noted the time commitment as one of the biggest challenges, sometimes suggesting that live interaction with other caregivers during group discussion weeks may have aided in overcoming this challenge. This suggests that, although many caregivers found value in the interactions present on the group discussion board, some may have preferred FF or traditional TH (where caregivers are physically present in the same place, but the therapist is connected with them remotely). However, the asynchronous group discussion board modality was developed to provide an additional option for families and clinicians who may not be able to access each other otherwise, due to travel, childcare, scheduling concerns, etc. Clinicians who wish to implement C-HOPE in the future should be mindful of family preferences and goodness of fit when considering modality of the intervention.

In addition to limitations of the modality/program, the modest sample size limits the ability to generalize findings. Future research with larger samples should examine whether engagement patterns predict changes in parent or child outcomes. Further, although all participating children had a confirmed community diagnosis of ASD, diagnostic tools were not re-administered as part of this study. Another limitation of the sample itself is the predominantly higher socio-economic status (SES) of the participants, which may reduce the generalizability of the findings to more diverse or underserved populations. While this study primarily included higher-income families, it is important to recognize that TH interventions, including asynchronous models, may have different levels of acceptability and effectiveness across diverse socio-economic and technologically literate populations. Research suggests that families from lower-income backgrounds or those with limited access to technology may face unique challenges in engaging with TH services (Crawford & Serhal, 2020). Caregiver satisfaction—defined as caregivers’ perceived usefulness and acceptability of an intervention—may be influenced by factors such as comfort with technology and access to digital resources (Pompa-Craven et al., 2022; Qu et al., 2022). Studies on TH interventions have suggested that underserved populations, such as those in rural areas or from lower-income families, may benefit from additional support in accessing and using asynchronous platforms (Uscher-Pines et al., 2020). As such, the generalizability of the current study’s findings to these groups may be limited. Future research should consider how adaptations to TH models can better accommodate diverse populations with varying levels of technological literacy.

Because the original dataset from this study is no longer accessible, we were unable to analyze whether outcomes differed based on concurrent behavioral or mental health service use or to examine potential differences between families who completed the intervention and those who did not. Given that over half of participating children were receiving other interventions during the study, this represents a limitation in our ability to evaluate potential differential or attrition effects. All outcome measures were based on caregiver report, and no independent observations or interobserver agreement data were collected. This reliance on caregiver-reported data limits the objectivity of the findings and should be addressed in future research through inclusion of direct behavioral observations. Further, ASD status was confirmed through parent report and completion of validated screening tools (M-CHAT-R/F or SCQ) rather than a standardized diagnostic assessment such as the ADOS-2, which may limit diagnostic precision.

The use of caregiver-reported fidelity may be limited by social desirability bias. Future research should ascertain fidelity of implementation from multiple sources. Additionally, engagement metrics were not available at the individual participant level, precluding analysis of whether higher levels of participation or time spent on Canvas were associated with greater outcome improvements. Caregiver comments drawn from the Canvas discussion boards are included to illustrate engagement and family perspectives; however, these naturally occurring reflections were not systematically coded or subjected to qualitative analysis and should be interpreted as illustrative rather than analytic data.

Further, the pre-post design itself is a limitation, as it cannot be said with confidence that decreases in child behavior noted by participants were due to participation in C-HOPE rather than some other factor(s). Thus, future research should include a large scale RCT with a diverse sample and follow-up evaluation post-intervention. Independent observation of child behavior change, made possible by store-and-forward videos if asynchronous modality is utilized, would also strengthen confidence in study results. Further, the study of an additional group modality—synchronous group discussion, where caregivers can interact with each other and with the facilitator in real time via videoconferencing, but from separate locations—is needed. The synchronous discussion group would function similarly to a live online class, where the facilitator would lead the group discussion just as typically done in FF and traditional TH modalities, and caregivers would be able to interact throughout the entire session. This change would address the participant comments made about the time commitment likely being more “worth it” if caregivers were able to interact with each other “live,” while also preserving the need for some families to avoid travel time and/or expenses.

In conclusion, caregivers of children with ASD need access to high-quality intervention services, and telehealth has the capacity to fill this very important need (Little et al., 2018a). The current study adapted a well-supported intervention, C-HOPE, to an asynchronous telehealth platform. Caregivers participated in online discussions and accessed recorded videos and digital materials through a popular learning management system. Caregivers who participated in this adaptation of C-HOPE reported reduction in behaviors and decreased parent stress, making it a viable intervention. This study shows that asynchronous telehealth interventions can be as successful as more traditional (i.e., in-person and synchronous telehealth sessions) autism interventions in reducing behaviors and parent stress.

## Figures and Tables

**Table 1:** Participant Demographic Information.

Child Variables		
	*M (SD)*	Range
Age in years	7.90 (2.25)	55–146
SCQ Total Score	26.67 (5.96)	14–35
	*n*	%
Race, as reported by parent (Black)	4	26.67%
Race, as reported by parent (White)	11	73.33%
Gender, as reported by parent (Female)	4	26.67%
Gender, as reported by parent (Male)	11	73.33%
Lives with mother only	3	20.00%
Lives with both mother and father	11	73.33%
Lives with other caregiver	1	6.67%
Receiving other behavioral or mental healthcare service	8	53.33%
**Family Variables**		
	*M (SD)*	Range
Mother years of education	15.80 (2.83)	12–21
Father years of education	14.15 (2.51)	12–18
Number of child’s siblings	1.47 (1.30)	0–4
	*n*	%
Parent participant gender, self-reported (Female)	15	100.00%
Residents of rural counties	4	26.67%
Annual household income (<$100,000 annual income)	9	60.00%

**Table 2:** C-HOPE Study Sessions and Topics Discussed.

Session	Duration	Format	Goals
Baseline Session	2 h	Online—Qualtrics	Assess all participants in the areas of child behavior, parenting stress, and parenting sense of competence; complete COMPASS profile
Individual Session 1	1 h	Telephone	Review COMPASS profile, identify a specific behavior to address; review C-HOPE philosophy, how to use data collection forms
Group Session 1	2 h	Online—Canvas	Overview of the program, ASD, resources, child behavior; introduction to and guided practice on progressive muscle relaxation
Group Session 2	2 h	Online—Canvas	Discuss child behavior, behavior management techniques; introduction to and guided practice on guided imagery
Individual Session 3	1 h	Telephone	Develop personalized behavior plan, including goals and objectives, using COMPASS framework
Group Session 3	2 h	Online—Canvas	Discuss parenting strategies, positive behavior management approaches; introduction to and guided practice on emotional grounding
Group Session 4	2 h	Online—Canvas	Discuss the emotions and stress associated with parenting a child with ASD
Individual Session 3	1 h	Telephone	Evaluate and modify behavior plan; review personal implementation of parenting strategies
Individual Session 4	1 h	Telephone/Online—Qualtrics	Review of program concepts; questions about implementation; assess all participants again in the areas of child behavior, parenting stress, and parenting sense of competence

*Note.* The original C-HOPE intervention (Kuravackel et al., 2018) included eight sessions (four 2 h group + four 1 h individual) delivered either face-to-face or via telehealth. The hybrid model retained the same session content and individual coaching structure but replaced live group meetings with asynchronous discussion boards moderated by the therapist.

**Table 3:** Paired Samples T-Tests for Main Outcome Measures.

Measure	Pre-Intervention		Post-Intervention		*t*(9)	*p*	Cohen’s *d*
	*M*	*SD*	*M*	*SD*			
BPS	55.20	17.59	50.50	17.51	1.11	0.30	0.27
ECBI	146.40	35.36	123.10	28.35	3.05	0.01	0.73
PSI-4	122.60	25.73	109.50	26.47	2.51	0.03	0.50

*Note.* For contextual comparison, the original C-HOPE study (Kuravackel et al., 2018) reported comparable pre- to post-improvements for child problem behavior (*d* = 0.73) and parent stress (*d* = 0.54), with smaller gains in parenting competence (*d* = 0.32). Values are included for descriptive reference only; no direct statistical comparison was conducted.

**Table 4:** Examples of discussion board prompts and responses.

GS#	Prompt	Sample Response(s)
1	Topic #2: The C-HOPE Triangle. How do you see the C-HOPE triangle (child behavior, parent stress, parenting sense of competency) playing out in your own life? In your child’s life?	Initial response: “My child’s behavior definitely plays a role in my stress level. It often determines where I go and who I am around when my child is with me. Most people don’t really understand him because they have a false sense of what ASD is suppose to look like. When he is having a bad day, I am rarely able to be consistent in my approach to discipline. Its like we walk on egg shells because we never really know how he is going to handle any given situation. I often feel like a bad parent when it comes to him because I find myself often just giving him what he wants to avoid further confrontations. My son can and will become violent, so I try to avoid making him frustrated but I know I dig myself in a deeper hole. This in turn stresses me out and cause me to become frustrated with him.” Another participant’s reply to this response: “My daughter’s challenges easily leave me feeling like I’m not doing/haven’t done enough, that somehow I should be able to engage her better and more consistently; even with behavioral strategies I feel like I don’t try hard enough, exercise the strategies consistently enough, stay ‘on my game’ enough. It starts to make me feel paralyzed sometimes.”
2	Topic #4: Replacement Behaviors. Given your unique child’s challenging behavior(s), what are some positive replacement behaviors/skills that you can identify? Looking at the Replacement Behaviors worksheet, what are some positive replacement behaviors you can identify for the specific behaviors listed?	Initial response: “A positive replacement behavior would be for [my son] to transition from outside to inside the house when we are all done with playing, without falling to the ground, because he does not want to come inside. So, I want to teach [my son] that he can walk inside the house and go to his room or his sensory room to have some quite time to relax and drop to the ground, nicely, instead of falling to the ground outside to avoid coming inside. This would still give him the input that he needs, falling to the ground, whatever that provides for him, and allow for a safer way to drop. This can become a safety issue when he decides he wants to stay on the neighborhood playground longer and tries to fall in the street, he doesn’t do it much, but has on occasion. I believe he has more of these types of behaviors when he is really sleepy.” Another participant’s reply to this response: “The sleepy part plays a big role with [my son] too! Nights he goes to bed earlier (like 8 p.m.) he is up at 5:30 a.m.- and his day at school does not go well. I think maybe he is tired before he evens gets there and certainly by end of school day!”
3	Topic #11: Emotional Grounding Which emotional grounding technique(s) do you think you might select to practice over the course of the next week as your relaxation exercise?	“I definitely use more of the soothing grounding. Especially when I am in the car going from place to place with fighting and screaming kids in the back seat or overwhelmed at work or at home, I always go to music or pray. I feel that this is more in the soothing category. It helps take my mind off of what is going on and then has me focus on positive images, songs, verses, etc. It completely takes me to a new place from where I was.”
4	Topic #4: Stages of Grief. Have you found yourself experiencing any of the stages of grief presented today? Have they followed a cyclical pattern, or are they more like a “rollercoaster” in your experience? What have you done to cope and to get yourself through any/all of the stages? Are there any stages that were presented which you have not experienced? Any you were surprised by?	“Yes, experienced these all & still go back through them as challenges & even smooth times occur. My denial was interesting because I wasn’t in denial about him having autism, I saw red flags & acted immediately. I limited my discussions with those that did not validate my concerns & we got him into [Early Intervention] quickly. My denial was when we started the process. I thought that because we were intervening early, that the therapists & teachers could ‘fix’ him & he would be ‘normal,’ maybe just a little quirky. I did not understand how this would eventually affect almost every part of our lives. How he would continue to be overwhelmed by small things, how he would need ongoing speech therapy & we still struggle to get or relay basic information to him, how it would take years to potty train, how he would continue to struggle to know how to play with toys, how his baby brother would pass him on certain skills, etc. So before I go on too much, yes, the stages of grief are alive & well because through this journey, I continue to uncover things that I need to grieve.”

## Data Availability

The datasets presented in this article are not readily available because of time limitations.

## Appendix A

### Group Session 1 Fidelity Checklist—Parent

**Instructions:** Below are the components of Group Session 1. Check the following boxes for the elements that occurred during the session.

1. **The therapist:**

- Described the goals of the session and an overview of upcoming sessions.
- Discussed the role that child problem behavior has on parent stress and parenting skills.
- Discussed the COMPASS model that says children can be competence when their challenges are balanced by supports.
- Reviewed expectations, roles, and confidentiality.
- Discussed how my child is similar and different from other children.
- Described what autism was and its possible causes.
- Described strategies for finding professionals and evaluating treatment options.
- Described theories about how my child thinks.
- Reviewed the ABC form that I used to collect information on my child’s behavior.
- Presented a relaxation strategy.
- Had me complete a satisfaction survey.
- Had me complete a rating scale about my thoughts of the group sessions.

2. **The session incorporated:**

- Activities to help understand what was taught (such as what how my child was unique but also similar to the other children, how my child thinks).
- Handouts for understanding about what autism was, its causes, and how to evaluate treatments.
- Facilitated guidance and structure from the therapist.
- A homework assignment to help me identify a goal for getting more services for my child or helping me to be aware of services that I might access in the future.

3. **The group:**

- Helped me feel supported.
- Helped me feel that I am not alone.
- Provided me with emotional support and to identify ways to promote my self-care and reduce my stress.
